# Associations of Different Types of Maternal Diabetes and Body Mass Index With Offspring Psychiatric Disorders

**DOI:** 10.1001/jamanetworkopen.2019.20787

**Published:** 2020-02-07

**Authors:** Linghua Kong, Ida A. K. Nilsson, Kerstin Brismar, Mika Gissler, Catharina Lavebratt

**Affiliations:** 1 Department of Molecular Medicine and Surgery, Karolinska Institutet, Stockholm, Sweden; 2 Translational Psychiatry Unit, Center for Molecular Medicine, Karolinska University Hospital, Stockholm, Sweden; 3 National Institute for Health and Welfare, Helsinki, Finland; 4 Division of Family Medicine, Department of Neurobiology, Care Sciences, and Society, Karolinska Institutet, Stockholm, Sweden; 5 Research Centre for Child Psychiatry, University of Turku, Turku, Finland

## Abstract

**Importance:**

Maternal obesity, pregestational type 1 and 2 diabetes, and gestational diabetes have been reported to increase the risk of autism spectrum disorder and attention-deficit/hyperactivity disorder in the mothers’ offspring. However, the associations of maternal diabetes disorders and body mass index jointly with psychiatric disorders among offspring are less well documented, especially for type 2 diabetes.

**Objective:**

To examine the associations of different types of maternal diabetes, separately and together with maternal obesity, with psychiatric disorders in the mothers’ offspring.

**Design, Setting, and Participants:**

This population-based cohort study used data from nationwide registries in Finland encompassing all 649 043 live births occurring between 2004 and 2014. The study and data analysis were conducted from January 1, 2019, to July 5, 2019.

**Exposures:**

Maternal prepregnancy body mass index, insulin-treated pregestational diabetes, and pregestational type 2 diabetes and gestational diabetes without insulin treatment.

**Main Outcomes and Measures:**

Psychiatric diagnoses and prescription of psychotropic drugs among the mothers’ offspring. Cox proportional hazards models were adjusted for birth year, sex, mode of delivery, maternal age, number of fetuses, parity, mother’s country of birth, mother’s marital status, maternal smoking, maternal psychiatric disorder, and maternal systemic inflammatory disease.

**Results:**

The mean (SD) age of mothers was 30.20 (5.37) years; 357 238 of 394 302 mothers (90.6%) were born in Finland. Of the 647 099 births studied, 4000 fetuses (0.62%) were exposed to maternal insulin-treated pregestational diabetes, 3724 (0.57%) were exposed to type 2 diabetes, and 98 242 (15.18%) were exposed to gestational diabetes; 34 892 offspring (5.39%) later received a diagnosis of a mild neurodevelopmental or psychiatric disorder. Non–insulin-treated type 2 diabetes in severely obese mothers, compared with normal-weight mothers without diabetes, was associated with psychiatric disorders in the offspring (hazard ratio, 1.97; 95% CI, 1.64-2.37), although with a lower effect size than that for severely obese mothers with insulin-treated pregestational diabetes (hazard ratio, 2.71; 95% CI, 2.03-3.61). The largest effect sizes were found for mood disorders, attention-deficit/hyperactivity disorder and conduct disorders, and autism. Gestational diabetes in severely obese mothers had a lower overall effect size (hazard ratio, 1.61; 95% CI, 1.50-1.72). Diabetes in normal-weight mothers was not associated with psychopathologic disorders in the offspring.

**Conclusions and Relevance:**

Severe obesity in mothers with diabetes was associated with an increased overall risk for psychiatric disorders in their offspring. The risk was highest for those exposed to insulin-treated pregestational diabetes, followed by non–insulin-treated type 2 diabetes and gestational diabetes. These findings may have implications for managing pregnancies.

## Key Points

**Question:**

Are different types of maternal diabetes, by themselves or in combination with maternal obesity, associated with an increased risk of offspring psychiatric disorders?

**Findings:**

In this cohort study of 647 099 births, non–insulin-treated pregestational type 2 diabetes in severely obese mothers was associated with several psychiatric disorders in their offspring, with an effect size lower than that for maternal insulin-treated pregestational diabetes but higher than that for gestational diabetes.

**Meaning:**

Maternal diabetes, mainly pregestational, in severely obese mothers may be associated with an excess risk of several psychiatric disorders in their offspring.

## Teaser

This population-based cohort study uses data from Finnish nationwide registries to examine the associations of different types of maternal diabetes, separately and together with maternal obesity, with psychiatric disorders in the mothers’ offspring.

## Introduction

Epidemiological and animal studies^1,2,3^ have demonstrated that prenatal exposure to maternal pregestational obesity is associated with neurodevelopmental and psychiatric disorders in offspring. In obesity, both metabolic and endocrine functions of adipose tissue are disturbed, with increased amounts of secreted proinflammatory cytokines, glycerol, nonesterified fatty acids, and hormones, thus increasing the risk of insulin resistance.^4,5,6,7,8^ Insulin resistance accompanied by deficient pancreatic insulin secretion leads to poor glucose control and increases the risk of type 2 diabetes.^6,7^ Moreover, type 1 diabetes, type 2 diabetes, and gestational diabetes can affect placental metabolic and endocrine functions, exposing the fetus^9^ to maternal hyperglycemia,^10^ lipotoxicity,^11^ oxidative stress,^12^ inflammation,^13,14,15^ and insulin resistance,^6,7^ which are thought to have long-lasting outcomes on organ development and function. The outcomes associated with hyperglycemia may, in part, be mediated by epigenetics.^16^

Furthermore, associations between in utero exposure to 3 main types of maternal diabetes and neurodevelopmental and psychiatric disorders among offspring have been reported.^10^ Specifically, fetal exposure to pregestational type 1 diabetes,^17^ type 2 diabetes, gestational diabetes,^18^ and nonspecified pregestational diabetes^19^ have been associated with the risk of autism spectrum disorder (ASD) in offspring. Also, a large multiethnic birth cohort study^20^ indicated that compared with no diabetes exposure, exposure to type 1 diabetes, type 2 diabetes, and gestational diabetes requiring antidiabetic medication were associated with the risk of offspring developing attention-deficit/hyperactivity disorder (ADHD) in a hierarchical order. Furthermore, a study^21^ stratified for maternal body mass index (BMI) (calculated as the weight in kilograms divided by height in meters squared) showed that the offspring of mothers with severe obesity with pregestational diabetes treated with insulin before and during pregnancy had a markedly increased risk of several psychiatric and mild neurodevelopmental disorders, whereas the presence of gestational diabetes did not markedly increase the risk for these disorders. However, mood and anxiety disorders, personality disorders, and intellectual disabilities were not studied adequately,^21^ and little is known about the risk for overweight, moderately obese, or severely obese mothers with type 2 diabetes to have offspring who develop mild neurodevelopmental variations and psychiatric conditions other than ASD.

Therefore, we aimed to explore whether and to what extent exposure to insulin-treated pregestational diabetes, non–insulin-treated type 2 diabetes, and gestational diabetes, stratified for maternal BMI, was associated with the risk of developing wide-spectrum psychiatric and neurodevelopmental disorders among offspring up to the age of 11 years using nationwide registries in Finland. We have previously reported associations of insulin-treated pregestational diabetes and gestational diabetes with some of the offspring psychiatric and neurodevelopmental disorders in this cohort, but we used a different reference group.^21^

## Methods

### Study Population and Data Sources

This population-based registry cohort study included all pregnancies ending in live births in Finland between January 1, 2004, and December 31, 2014 (649 043 births), obtained from the Drugs and Pregnancy database.^22^ The Drugs and Pregnancy database steering committee and the data protection authority in Finland approved this study. Informed consent is not required in Finland for this type of study, because no participants were contacted. This study follows the Strengthening the Reporting of Observational Studies in Epidemiology (STROBE) reporting guideline. The study and data analysis were conducted from January 1, 2019, to July 5, 2019.

The data from the Drugs and Pregnancy database^22^ were derived from the Medical Birth Register, the Register of Congenital Malformations, and the Register on Induced Abortions. The data on maternal and offspring drug purchases were obtained from the Finnish Register on Reimbursement Drugs. All medicines considered were prescription only: prescribed by a physician or dentist and dispensed solely in pharmacies. All permanent Finnish residents and citizens are entitled to reimbursement of prescribed medicine. All medicines for offspring and mothers were identified from reimbursement of drug-purchasing costs. Offspring and maternal medical diagnoses were extracted from the Finnish Care Registers for Health Care (HILMO) (eAppendix in the Supplement). Data from the different registers were linked using unique personal identification numbers assigned to all permanent residents and Finnish citizens and recorded in all registers (National Institute for Health and Welfare and National Social Insurance Institution).

### Main Exposures

Data on prepregnancy BMI, recorded at the first prenatal visit (gestational weeks 7-10), were obtained from the Drugs and Pregnancy database. The use of BMI restricted the inclusion to 2004 at the earliest. Maternal BMI was categorized as severely obese (≥35), moderately obese (≥30 to <35), overweight (≥25 to <30), and normal weight (≥18.5 to <25).

In Finland, there is specialist care for women with pregestational and gestational diabetes with the goal of achieving close to normal blood glucose levels. Pregestational diabetes along with pregestational purchase of insulin (hereafter referred to as *insulin-treated pregestational diabetes*) was identified from the Finnish Register on Reimbursement Drugs, which records the special reimbursement of insulin medication for diabetes (to which to all Finnish citizens and permanent residents are entitled). For the mothers without insulin-treated pregestational diabetes, diagnoses of type 2 diabetes and gestational diabetes were identified from HILMO and the Finnish Register on Reimbursement Drugs. Mothers with type 2 diabetes were those with at least 1 of the *International Statistical Classification of Diseases and Related Health Problems, Tenth Revision (ICD-10)* diagnosis codes O24.1, E11, and E14 before pregnancy, and/or a purchase of a Anatomic Therapeutic Chemical (ATC) group A10B drug (ie, blood glucose–lowering drugs other than insulin) before pregnancy. Gestational diabetes was identified through *ICD-10* diagnosis code O24.4. Those without insulin-treated pregestational diabetes, type 2 diabetes, or gestational diabetes were grouped as not having diabetes. Mothers with a purchase of insulin during pregnancy were excluded from the type 2 diabetes, gestational diabetes, and no diabetes groups (16 with type 2 diabetes, 326 with gestational diabetes, and 1602 without diabetes), and similarly, mothers with type 2 diabetes were excluded from the gestational diabetes group. In total, 647 099 births were included in the 4 diabetes exposure categories studied here. Registers are described in the eAppendix in the Supplement.

### Outcomes and Covariates

Information on psychiatric disorders, as primary or secondary diagnoses, for offspring and mothers was obtained from HILMO. Among offspring, the diagnosis groups indicated by the following *ICD-10* codes were examined as outcome variables: F30 through F39 and F92; F40 through F43 and F93; and F50, F51, F60 through F69, F70 through F79, F80 through F83, F84, F90 and F91, and F98 (Table 1). The grouping of diagnoses was based on symptom similarities and was implemented to enhance the statistical power of the analyses. Because cases with F50 or F84 diagnoses were not found in the 2014 birth cohort, and cases within the diagnosis groups F30 through F39 and F92, F40 through F43 and F93, F60 through F69, F70 through F79, and F90 and F91 were not found in the 2013 to 2014 birth cohorts, those cohorts were excluded from the analyses of these respective groups.

**Table 1.** The *ICD-10* Codes and Corresponding Psychiatric and Mild Neurodevelopmental Disorders and Age at Onset for the 2004 to 2014 Birth Cohort

*ICD-10* Codes	Psychiatric and Neurodevelopmental Disorders	Cases Estimated to Be Diagnosed Before Age 19 y, %^a^	Age at Onset, Median (IQR), y
F30-F39, F92	Mood disorders	31.7	6.7 (5.1-8.1)
F40-F43, F93	Anxiety disorders	39.8	5.8 (4.2-7.5)
F50	Eating disorders	28.6	2.7 (1.0-5.9)
F51	Sleeping disorders	55.5	1.0 (0.7-1.9)
F60-F69	Personality disorders	4.7	5.5 (3.4-7.9)
F70-F79	Intellectual disabilities	27.3	3.7 (2.5-5.4)
F80-F83	Specific developmental disorders	27.0	1.0 (0.7-1.9)
F84	Autism spectrum disorder	57.2	3.9 (2.9-5.7)
F90-F91	Attention-deficit/hyperactivity disorder and conduct disorders	61.2	6.1 (4.7-7.6)
F98	Other behavioral and emotional disorders	76.2	4.6 (0.6-6.1)

Abbreviations: *ICD-10*, *International Statistical Classification of Diseases and Related Health Problems, Tenth Revision*; IQR, interquartile range.

^a^ Proportion identified of the number of cases estimated to be diagnosed before age 19 years. This number was estimated as 16 or 18 times the number of cases in the 1996 birth cohort, which was followed up until 2014 (16 times for diagnosis groups studied in the 1996-2012 cohorts, and 18 times for the diagnoses studied in the 1996-2014 cohorts).

Data on purchase of psychotropic drugs for offspring were extracted from the Finnish Register on Reimbursement Drugs and applied as outcome variables in a second model. The following ATC codes were used: N05 (antipsychotics, anxiolytics, hypnotics, and sedatives), N06A (antidepressants), and N06B (psychostimulants and nootropics). Also, data on mothers’ previous inpatient care associated with pregestational mental health disorders (*International Classification of Diseases, Eighth Revision* codes 290-317 in 1969-1986, *International Classification of Diseases, Ninth Revision* codes 290-319 in 1987-1995, and *ICD-10* codes F00-F99 in 1996-2014 from HILMO) and purchase of ATC group N05 or N06 medications during pregnancy were used as covariates.

Information on offspring birth year, sex, any perinatal problem (birth weight <2500 g or gestational age <37 weeks, or small birth weight according to gestational age according to Finnish sex-specific standards^23,24^), number of fetuses, mode of delivery, maternal age at delivery, parity, maternal marital status, mother’s country of birth, and maternal smoking were obtained from the Drugs and Pregnancy database. Information on mothers’ diagnoses of systemic inflammatory disorders (*ICD-10* codes M30-M36 in 2004-2014) as primary or secondary diagnoses was obtained from HILMO. The *ICD-10* was in routine use over the period between 1996 and 2014. Maternal and birth characteristics of the cohort studied are shown in eTable 1 in the Supplement.

### Statistical Analysis

**Figure 1.**
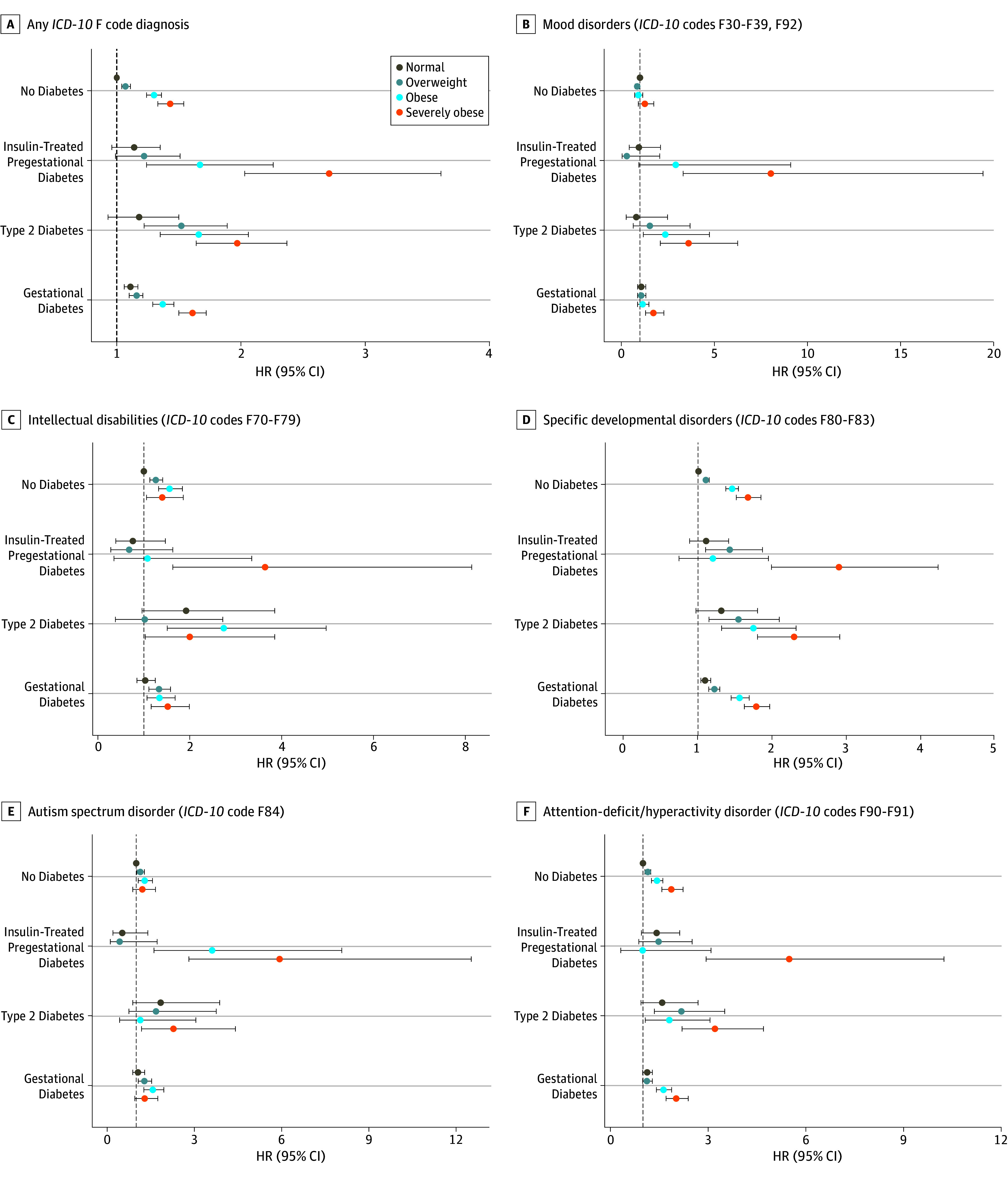
Risks of Psychiatric and Mild Neurodevelopmental Disorders in Offspring According to Maternal Body Mass Index and Diabetes For 647 099 births, graphs show adjusted hazard ratios (HRs) with error bars representing 95% CIs for any *International Statistical Classification of Diseases and Related Health Problems, Tenth Revision (ICD-10) *F code diagnosis (A), mood disorders (B), intellectual disabilities (C), specific developmental disorders (D), autism spectrum disorder (E), and attention-deficit/hyperactivity disorder or conduct disorders (F). The analyses were adjusted for offspring birth year, sex, perinatal problems, number of fetuses, cesarean delivery, maternal age group at delivery, parity, mother’s marital status at birth, mother’s country of birth, maternal smoking, maternal psychiatric disorder, maternal use of psychotropic medication during pregnancy (Anatomic Therapeutic Chemical group N05 or N06 drugs), and maternal systemic inflammatory disease. Body mass index (calculated as the weight in kilograms divided by height in meters squared) was categorized as severely obese (≥35), obese (≥30 to <35), overweight (≥25 to <30), and normal (≥18.5 to <25).

**Figure 2.**
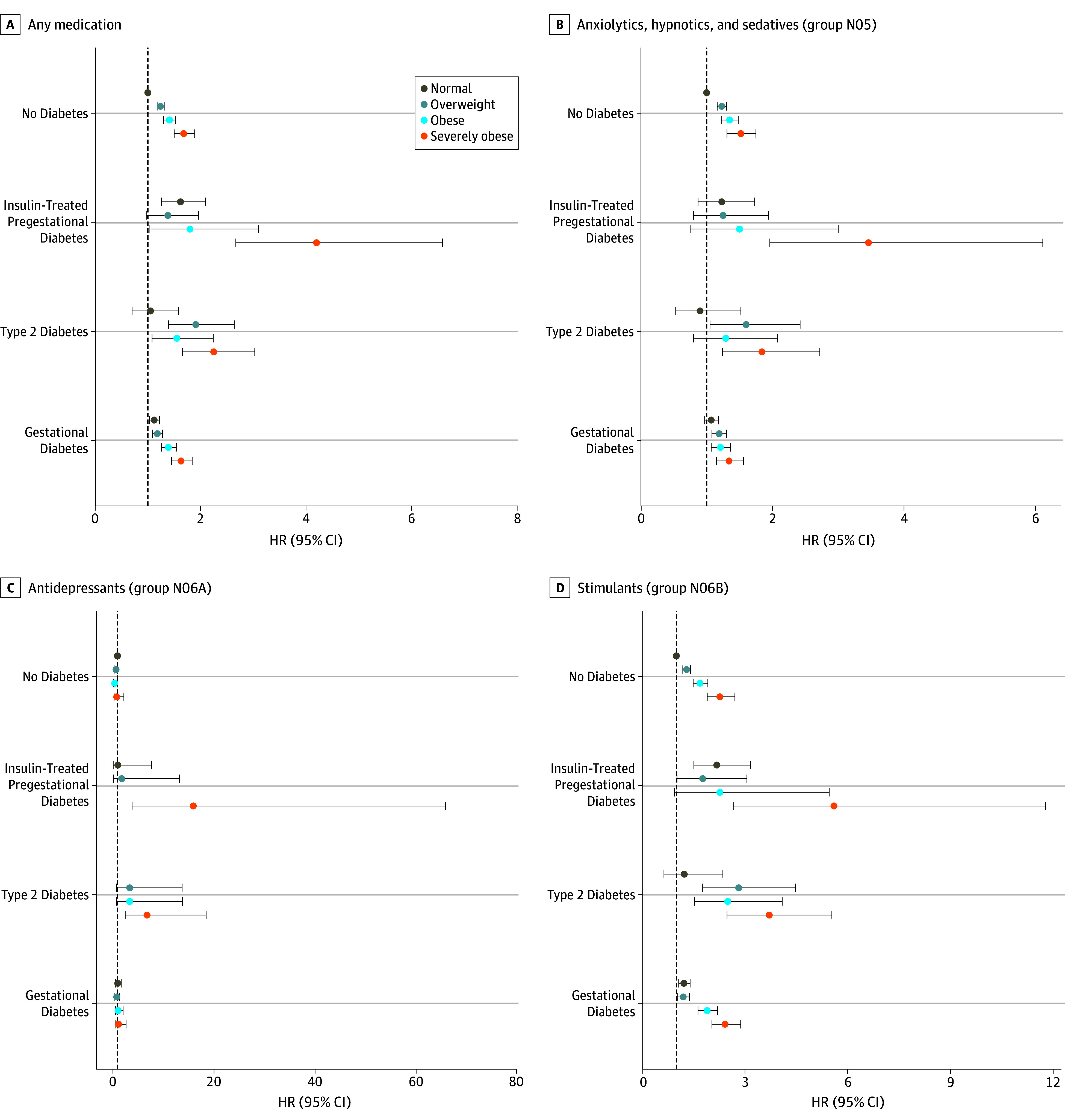
Risks of Offspring Receiving Psychotropic Medications According to Maternal Body Mass Index and Diabetes For 647 099 births, graphs show adjusted hazard ratios (HRs) with error bars representing 95% CIs for offspring receiving any psychotropic medication (A); anxiolytics, hypnotics, and sedatives (B); antidepressants (C); and stimulants (D). The analyses were adjusted for offspring birth year, sex, perinatal problems, number of fetuses, cesarean delivery, maternal age group at delivery, parity, mother’s marital status at birth, mother’s country of birth, maternal smoking, maternal psychiatric disorder, maternal use of psychotropic medication during pregnancy (Anatomic Therapeutic Chemical groups N05 and N06), and maternal systemic inflammatory disease. Body mass index (calculated as the weight in kilograms divided by height in meters squared) was categorized as severely obese (≥35), obese (≥30 to <35), overweight (≥25 to <30), and normal (≥18.5 to <25).

Cox proportional hazards modeling was used to estimate the association of exposure to maternal prepregnancy obesity and different types of diabetes, the latter stratified by BMI categories, with the outcomes offspring psychiatric diagnosis and purchase of psychotropic drug (sensitivity analysis). For maternal BMI, the strata overweight, moderately obese, and severely obese were compared with normal weight. Covariates were adjusted for as indicated in Figure 1, Figure 2, Table 2, and Table 3. Hazard ratios (HRs) with 95% CIs were reported as measures of effect size. Two-sided *P* < .05 was considered statistically significant. All statistical analyses were performed using SAS statistical software version 9.3 (SAS Institute).

**Table 2.** Hazard Ratios for Offspring Psychiatric and Mild Neurodevelopmental Disorders According to Maternal BMI and Diabetes^a^

Diabetes Categories, by BMI^b^	HR (95% CI)										
	Any *ICD-10* F Code Diagnosis (n = 34 892)	Mood Disorders (n = 1999)	Anxiety Disorders (n = 4713)	Eating Disorders (n = 279)	Sleeping Disorders (n = 2 219)	Personality Disorders (n = 175)	Intellectual Disabilities (n = 2507)	Specific Developmental Disorders (n = 17 923)	ASD (n = 2346)	ADHD and Conduct Disorders (n = 5263)	Other Disorders (n = 8506)
No diabetes (n = 541 133)											
18.5 to <25	1 [Reference]	1 [Reference]	1 [Reference]	1 [Reference]	1 [Reference]	1 [Reference]	1 [Reference]	1 [Reference]	1 [Reference]	1 [Reference]	1 [Reference]
≥25 to <30	1.07 (1.04-1.11)	0.85 (074-0.98)	0.96 (0.88-1.05)	1.00 (0.71-1.41)	1.07 (0.96-1.21)	0.88 (0.56-1.39)	1.26 (1.13-1.41)	1.10 (1.06-1.15)	1.14 (1.02-1.28)	1.15 (1.06-1.24)	1.01 (0.94-1.07)
≥30 to <35	1.30 (1.24-1.36)	0.91 0.72-1.15)	1.31 (1.16-1.49)	0.81 (0.43-1.53)	1.00 (0.82-1.22)	2.08 (1.23-3.52)	1.56 (1.32-1.84)	1.46 (1.37-1.55)	1.29 (1.07-1.56)	1.43 (1.26-1.61)	1.13 (1.03-1.26)
≥35	1.43 (1.33-1.54)	1.26 (0.91-1.74)	1.41 (1.15-1.72)	0.89 (0.33-2.40)	1.04 (0.75-1.42)	0.69 (0.17-2.81)	1.40 (1.06-1.86)	1.67 (1.52-1.84)	1.21 (0.88-1.66)	1.87 (1.58-2.23)	1.14 (0.97-1.35)
Insulin-treated pregestational diabetes (n = 4000)											
18.5 to <25	1.14 (0.96-1.35)	0.94 (0.42-2.10)	1.01 (0.61-1.69)	2.59 (0.82-8.26)	0.99 (0.47-2.10)	NA	0.76 (0.39-1.47)	1.11 (0.88-1.41)	0.52 (0.20-1.40)	1.42 (0.95-2.13)	0.98 (0.69-1.38)
≥25 to <30	1.22 (0.99-1.551)	0.29 (0.04-2.06)	0.83 (0.40-1.75)	NA	2.29 (1.89-4.43)	NA	0.68 (0.28-1.63)	1.43 (1.10-1.87)	0.43 (0.11-1.72)	1.48 (0.87-2.51)	1.07 (0.69-1.64)
≥30 to <35	1.67 (1.24-2.26)	2.92 (0.94-9.10)	1.78 (0.74-4.28)	3.81 (0.53-27.57)	2.31 (0.86-6.17)	NA	1.08 (0.35-3.35)	1.21 (0.75-1.95)	3.61 (1.61-8.07)	0.99 (0.32-3.09)	1.89 (1.12-3.20)
≥35	2.71 (2.03-3.61)	8.03 (3.32-19.43)	1.05 (0.26-4.18)	NA	1.48 (0.37-5.94)	NA	3.64 (1.63-8.14)	2.91 (1.99-4.24)	5.93 (2.81-12.52)	5.49 (2.94-10.24)	2.93 (1.76-4.87)
Type 2 diabetes (n = 3724)											
18.5 to <25	1.18 (0.93-1.50)	0.80 (0.26-2.48)	1.15 (0.62-2.14)	2.00 (0.28-14.33)	0.60 (0.15-2.40)	NA	1.92 (0.96-3.85)	1.32 (0.97-1.81)	1.84 (0.88-3.87)	1.59 (0.94-2.69)	0.98 (0.58-1.65)
≥25 to <30	1.52 (1.22-1.89)	1.53 (0.64-3.69)	1.29 (0.69-2.40)	NA	0.66 (0.16-2.63)	NA	1.02 (0.38-2.72)	1.55 (1.15-2.10)	1.68 (0.75-3.75)	2.18 (1.35-3.51)	1.41 (0.89-2.24)
≥30 to <35	1.66 (1.35-2.06)	2.36 (1.18-4.73)	1.52 (0.86-2.68)	2.14 (0.30-15.34)	1.62 (0.67-3.91)	NA	2.74 (1.51-4.97)	1.75 (1.32-2.33)	1.14 (0.43-3.05)	1.81 (1.07-3.06)	1.57 (1.01-2.44)
≥35	1.97 (1.64-2.37)	3.61 (2.09-6.25)	2.15 (1.35-3.42)	1.96 (0.27-14.03)	NA	NA	2.00 (1.03-3.85)	2.29 (1.81-2.91)	2.28 (1.18-4.41)	3.21 (2.20-4.70)	1.65 (1.10-2.46)
Gestational diabetes (n = 98 242)											
18.5 to <25	1.11 (1.06-1.17)	1.07 (0.87-1.31)	0.98 (0.85-1.12)	1.33 (0.82-2.16)	1.21 (1.01-1.44)	0.60 (0.24-1.48)	1.03 (0.85-1.25)	1.10 (1.03-1.18)	1.06 (0.88-1.29)	1.13 (0.99-1.29)	1.18 (1.07-1.30)
≥25 to <30	1.16 (1.10-1.21)	1.07 (0.87-1.32)	1.06 (0.92-1.21)	0.62 (0.31-1.27)	0.95 (0.78-1.17)	0.95 (0.46-1.95)	1.33 (1.11-1.58)	1.22 (1.14-1.30)	1.28 (1.07-1.53)	1.13 (0.99-1.29)	1.11 (1.00-1.22)
≥30 to <35	1.37 (1.29-1.46)	1.14 (0.87-1.48)	1.22 (1.04-1.44)	0.83 (0.37-1.87)	0.75 (0.55-1.01)	1.23 (0.54-2.81)	1.34 (1.07-1.68)	1.57 (1.45-1.70)	1.57 (1.26-1.95)	1.63 (1.41-1.88)	1.24 (1.10-1.40)
≥35	1.61 (1.50-1.72)	1.72 (1.30-2.28)	1.47 (1.21-1.79)	1.35 (0.60-3.04)	0.88 (0.62-1.24)	0.68 (0.17-2.76)	1.52 (1.16-1.99)	1.79 (1.63-1.97)	1.29 (0.95-1.74)	2.02 (1.71-2.39)	1.33 (1.14-1.54)

Abbreviations: ADHD, attention-deficit/hyperactivity disorder; ASD, autism spectrum disorders; BMI, body mass index (calculated as the weight in kilograms divided by height in meters squared); HR, hazard ratio; *ICD-10*, *International Statistical Classification of Diseases and Related Health Problems, Tenth Revision*; NA, not applicable.

^a^ Data are shown for 647 099 births. For *ICD-10* codes F51 (sleeping disorders), F80 to F83 (specific developmental disorders), and F98 (other behavioral and emotional disorders), the birth cohorts for 2004 to 2014 were used; for *ICD-10* codes F50 (eating disorders) and F84 (ASD), the birth cohorts for 2004 to 2013 were used; and for *ICD-10* codes F30 to F39 and F92 (mood disorders), F40 to F43 and F93 (anxiety disorders), F60 to F69 (personality disorders), F70 to F79 (intellectual disabilities), and F90 to F91 (ADHD and conduct disorders), the birth cohorts for 2004 to 2012 were used. All were followed up until 2014. Type 2 diabetes was defined as pregestational type 2 diabetes (*ICD-10* code E11, E14, or O24.1 and/or the use of Anatomic Therapeutic Chemical class A10B drugs) without insulin purchase before or during pregnancy. Gestational diabetes was defined as an *ICD-10* code O24.4 diagnoses without purchase of insulin before or during pregnancy. Insulin-treated pregestational diabetes was defined as pregestational diabetes with the purchase of insulin before pregnancy.

^b^ The analyses were adjusted for offspring birth year, sex, perinatal problems, number of fetuses, cesarean delivery, maternal age group at delivery, parity, mother’s marital status at birth, mother’s country of birth, maternal smoking, maternal psychiatric disorder, maternal use of psychotropic medication during pregnancy (Anatomic Therapeutic Chemical class N05 or N06 drugs), and maternal systemic inflammatory disease. The reference is births to normal-weight mothers without diabetes and without purchase of insulin. Maternal BMI was categorized as severely obese (≥35), obese (≥30 to <35), overweight (≥25 to <30), and normal (18.5 to <25).

**Table 3.** Hazard Ratios for Offspring Psychotropic Medications According to Maternal BMI and Diabetes^a^

Diabetes Categories, by BMI^b^	HR (95% CI)			
	Any Medication (n = 13 436)	Anxiolytics, Hypnotics, and Sedatives (n = 9445)	Antidepressants (n = 334)	Stimulants (n = 4613)
No diabetes (n = 541 133)				
18.5 to <25	1 [Reference]	1 [Reference]	1 [Reference]	1 [Reference]
≥25 to <30	1.24 (1.19-1.31)	1.23 (1.16-1.30)	0.72 (0.50-1.04)	1.30 (1.19-1.41)
≥30 to <35	1.41 (1.30-1.52)	1.35 (1.23-1.48)	0.45 (0.20-1.00)	1.69 (1.49-1.92)
≥35	1.68 (1.50-1.89)	1.52 (1.31-1.75)	0.85 (0.31-2.28)	2.27 (1.90-2.71)
Insulin-treated pregestational diabetes (n = 4000)				
18.5 to <25	1.62 (1.26-2.09)	1.23 (0.87-1.73)	1.08 (0.15-7.78)	2.18 (1.51-3.16)
≥25 to <30	1.38 (0.97-1.96)	1.25 (0.80-1.94)	1.84 (0.25-13.30)	1.77 (1.02-3.06)
≥30 to <35	1.80 (1.04-3.10)	1.50 (0.75-3.00)	NA	2.27 (0.94-5.46)
≥35	4.20 (2.67-6.59)	3.46 (1.96-6.11)	16.00 (3.88-65.94)	5.60 (2.66-11.77)
Type 2 diabetes (n = 3724)				
18.5 to <25	1.05 (0.70-1.58)	0.90 (0.53-1.52)	NA	1.23 (0.64-2.36)
≥25 to <30	1.91 (1.39-2.64)	1.60 (1.05-2.42)	3.42 (0.85-13.80)	2.82 (1.77-4.48)
≥30 to <35	1.55 (1.08-2.24)	1.29 (0.80-2.08)	3.42 (0.85-13.85)	2.50 (1.53-4.09)
≥35	2.25 (1.66-3.03)	1.84 (1.24-2.72)	6.85 (2.53-18.54)	3.71 (2.48-5.54)
Gestational diabetes (n = 98 242)				
18.5 to <25	1.12 (1.03-1.22)	1.07 (0.97-1.18)	1.06 (0.65-1.73)	1.22 (1.07-1.40)
≥25 to <30	1.18 (1.09-1.28)	1.19 (1.08-1.30)	0.85 (0.49-1.45)	1.20 (1.04-1.38)
≥30 to <35	1.39 (1.26-1.54)	1.21 (1.07-1.36)	1.12 (0.60-2.12)	1.90 (1.63-2.20)
≥35	1.63 (1.45-1.84)	1.34 (1.15-1.56)	1.20 (0.53-2.70)	2.42 (2.04-2.88)

Abbreviations: ATC, Anatomic Therapeutic Chemical; BMI, body mass index (calculated as the weight in kilograms divided by height in meters squared); HR, hazard ratio; *ICD-10*, *International Statistical Classification of Diseases and Related Health Problems, Tenth Revision*; NA, not applicable.

^a^ Data are shown for 647 099 births. Psychotropic medications were defined according to the ATC classification system: antipsychotics, anxiolytics, hypnotics, and sedatives (ATC group N05); antidepressants (ATC group N06A); and stimulants (ATC group N06B). For *ICD-10* codes F51 (sleeping disorders), F80 to F83 (specific developmental disorders), and F98 (other behavioral and emotional disorders), the birth cohorts 2004 to 2014 were used; for *ICD-10* codes F50 (eating disorders) and F84 (autism spectrum disorder), the birth cohorts 2004 to 2013 were used; and for *ICD-10 *codes F30 to F39 and F92 (mood disorders), F40 to F43 and F93 (anxiety disorders), F60 to F69 (personality disorders), F70 to F79 (intellectual disabilities), and F90 to F91 (attention-deficit/hyperactivity disorder and conduct disorders), the birth cohorts 2004 to 2012 were used. All were followed up until 2014. Insulin-treated pregestational diabetes was defined as pregestational diabetes with purchase of insulin before pregnancy. Type 2 diabetes was defined as pregestational type 2 diabetes (*ICD-10* code E11, E14, or O24.1 and/or ATC group A10B) without insulin purchase before or during pregnancy. Gestational diabetes was defined as an *ICD-10* code O24.4 diagnoses without purchase of insulin before or during pregnancy.

^b^ The analyses were adjusted for offspring birth year, sex, perinatal problems, number of fetuses, cesarean delivery, maternal age group at delivery, parity, mother’s marital status at birth, mother’s country of birth, maternal smoking, maternal psychiatric disorder, maternal use of psychotropic medication during pregnancy (ATC group N05 or N06), and maternal systemic inflammatory disease. The reference is births to normal-weight mothers without diabetes and without purchase of insulin. Maternal BMI was categorized as severely obese (≥35), obese (≥30 to <35), overweight (≥25 to <30), and normal (18.5 to <25).

In a sensitivity analysis, the first 2 subsequent singleton pregnancies of the same mother during the study period were included (247 681 pregnancies). The first child born during the study period was not necessarily the first child born to the mother. The exposures for maternal diabetes (eTable 2 in the Supplement) and BMI (eTable 3 in the Supplement) for the 2 offspring were recorded. Because only a few sibling pairs were discordant for a maternal diabetes category, exposure-discordant sibling pair analysis could not be performed. Instead, the risk for the outcome in the second offspring given maternal diabetes exposure was estimated irrespectively of the older sibling (model 1). In an attempt to adjust for familial risk of the outcome, the outcome for the older sibling was also followed up for psychiatric diagnoses or psychotropic medication until 2014. In model 2, we adjusted the second offspring’s outcome risk by the outcome in the older sibling irrespective of the maternal diabetes and BMI exposure for the older sibling.

## Results

The mean (SD) age of mothers was 30.20 (5.37) years, and 357 238 of 394 302 mothers (90.6%) were born in Finland. Of the 647 099 births studied, 34 892 offspring (5.39%) had a psychiatric disorder (ie, any *ICD-10* F code) diagnosed between 2004 and 2014—that is, up to the age of 11 years for the oldest children (median [interquartile range], 5.5 [2.8-8.3] years). Of the 647 099 children, 1999 (0.31%) had mood disorders, 4713 (0.73%) had anxiety disorders, 279 (0.043%) had eating disorders, 2219 (0.34%) had sleeping disorders, 175 (0.027%) had personality disorders, 2507 (0.39%) had intellectual disabilities, 17 923 (2.77%) had specific developmental disorders, 2346 (0.36%) had ASD, 5263 (0.81%) had ADHD or conduct disorders, and 6976 (1.31%) had other behavioral and emotional disorders. We examined the proportion of cases identified of those estimated to receive a diagnosis before age 19 years using the diagnosis rates in the 1996 birth cohort. Between 27.0% and 39.8% of cases were detected for mood disorders, anxieties, eating disorders, intellectual disabilities, and specific developmental disorders, whereas 55.5% to 76.2% of cases were detected for sleeping disorders, ASD, ADHD, and conduct disorders, and other behavioral and emotional disorders (*ICD-10* code F98) (Table 1).

A purchase of psychotropic medication was recorded for 13 436 children (2.07%), including antipsychotics and hypnotics or anxiolytics (9445 ATC group N05 drugs), antidepressants (334 ATC group N06A drugs), and stimulants (4613 ATC group N06B drugs). Maternal pregestational BMI was normal for 59.2% of births, overweight for 20.7%, moderately obese for 7.7%, and severely obese for 3.7%. Furthermore, 105 966 fetuses (16.3%) were exposed in utero to maternal diabetes, 4000 (0.62%) to insulin-treated pregestational diabetes (3880 with insulin purchase also during pregnancy), 3724 (0.57%) to type 2 diabetes not treated with insulin (3349 [90%] had an *ICD-10* code O24.1 or E11 diagnosis), and 98 242 (15.18%) to gestational diabetes not treated with insulin. The purchase of glucose-lowering drugs other than insulin during pregnancy was not adjusted for because only 0.38% of mothers with insulin-treated pregestational diabetes, 0.43% of mothers with type 2 diabetes, 0.32% of mothers with gestational diabetes, and 0.28% of mothers without diabetes purchased such drugs. eTable 1 in the Supplement shows additional characteristics of mothers and offspring.

First, among mothers without diabetes, higher maternal prepregnancy BMI (moderate and severe obesity) was associated with an increased risk of any *ICD-10* F code diagnosis among their offspring (moderate obesity, HR, 1.30 [95% CI, 1.24-1.36]; severe obesity, HR, 1.43 [95% CI, 1.33-1.54]), specifically anxiety disorders (*ICD-10* codes F40-F43 and F93; moderate obesity, HR, 1.31 [95% CI, 1.16-1.49]; severe obesity, HR, 1.41 [95% CI, 1.15-1.72]), intellectual disabilities (*ICD-10* codes F70-F79; moderate obesity, HR, 1.56 [95% CI, 1.32-1.84]; severe obesity, HR, 1.40 [9% CI, 1.06-1.86]), specific developmental disorders (*ICD-10* codes F80-F83; moderate obesity, HR, 1.46 [95% CI, 1.37-1.55]; severe obesity, HR, 1.67 [95% CI, 1.52-1.84]), and ADHD or conduct disorders (*ICD-10* codes F90-F91; moderate obesity, HR, 1.43 [95% CI, 1.26-1.61]; severe obesity, HR, 1.87 [95% CI, 1.58-2.23]) compared with mothers with BMI less than 25, where associations with the latter 2 diagnoses groups were reported from these birth cohorts previously^21^ (Figure 1 and Table 2).

Second, there were statistically significant interactions between maternal prepregnancy BMI and maternal diabetes with offspring having any *ICD-10* F code diagnosis (insulin-treated pregestational diabetes, *P* for interaction < .001; type 2 diabetes, *P* for interaction < .001; gestational diabetes, *P* for interaction = .007). Therefore, the associations between maternal diabetes and offspring disorders were assessed after stratifying for maternal prepregnancy BMI. Maternal diabetes implied, on top of obesity, an additional increased risk for offspring to receive any *ICD-10* F code diagnosis, with the pointwise effect size generally larger in the order insulin-treated pregestational diabetes first, then type 2 diabetes, and then gestational diabetes (Figure 1). Maternal insulin-treated pregestational diabetes (HR, 2.71; 95% CI, 2.03-3.61), type 2 diabetes (HR, 1.97; 95% CI, 1.64-2.37), and gestational diabetes (HR, 1.61; 95% CI, 1.50-1.72) implied an estimated 128%, 54%, and 18% increased risk for offspring to receive any *ICD-10* F code diagnosis among mothers with severe obesity compared with mothers with severe obesity only (HR, 1.43; 95% CI, 1.33-1.54), with the reference being normal-weight mothers without diabetes (Figure 1A and Table 2). Severe obesity in insulin-treated pregestational diabetes was associated with offspring mood disorders (HR, 8.03; 95% CI, 3.32-19.43) and intellectual disability (HR, 3.64; 95% CI, 1.63-8.14) with large effect sizes (Figure 1B and C and Table 2), although there was no association with offspring anxiety disorders (HR, 1.05; 95% CI, 0.26-4.18) (Table 2). Previously, we reported that the offspring of severely obese mothers who had insulin-treated pregestational diabetes had a pointwise 6-time increased risk of ASD and ADHD, but the nondiabetic reference group included type 2 diabetes.^21^ For type 2 diabetes, all data in the present study are novel. The offspring of severely obese mothers with type 2 diabetes had higher risks of mood disorders (HR, 3.61; 95% CI, 2.09-6.25), anxiety disorders (HR, 2.15; 95% CI, 1.35-3.42), intellectual disabilities (HR, 2.00; 95% CI, 1.03-3.85), specific developmental disorders (HR, 2.29; 95% CI, 1.81-2.91), ASD (HR, 2.28; 95% CI, 1.18-4.41), ADHD or conduct disorders (HR, 3.21; 95% CI, 2.20-4.70), and other behavioral and emotional disorders (HR, 1.65; 95% CI, 1.10-2.46) compared with the offspring of normal-weight mothers without diabetes. Moreover, these risks for mood disorders and ADHD or conduct disorders were higher than those for the offspring of severely obese mothers without diabetes (mood disorders, HR, 1.26; 95% CI, 0.91-1.74; ADHD or conduct disorders, HR, 1.87; 95% CI, 1.58-2.23). Exposure to gestational diabetes was not associated with any specific *ICD-10* F code diagnosis. As for insulin-treated pregestational diabetes, data for gestational diabetes were not previously reported for mood, anxiety, or personality disorders or intellectual disabilities.^21^ Thus, among mothers with severe obesity, the effect sizes were lower for type 2 diabetes than for insulin-treated pregestational diabetes in association with offspring mood disorders, specific developmental disorders, ASD, ADHD or conduct disorders, and other behavioral and emotional disorders. Compared with severely obese mothers with gestational diabetes, the effect sizes for severely obese mothers with type 2 diabetes were higher for mood disorders, specific developmental disorders, ASD, and ADHD or conduct disorders (Figure 1 and Table 2).

Third, in a sensitivity analysis, we used the purchase of psychotropic medication, including ATC group N05 drugs (antipsychotics, anxiolytics, hypnotics, and sedatives), group N06A drugs (antidepressants), and group N06B drugs (stimulants and nootropics), as an estimate of offspring psychiatric disorders. The findings for offspring of mothers with type 2 diabetes are novel, but the results for the offspring of mothers with insulin-treated pregestational diabetes and gestational diabetes were reported previously^21^ with a nondiabetic reference group including type 2 diabetes (Table 3 and Figure 2). Similar to the analysis results where psychiatric diagnoses were outcome variables, offspring of severely obese mothers with insulin-treated pregestational diabetes or type 2 diabetes had the highest effect size on psychotropic medication purchase. The effect size for any purchase among offspring to severely obese mothers with type 2 diabetes (HR, 2.25; 95% CI, 1.66-3.03) was higher than that for severely obese mothers without diabetes (HR, 1.68; 95% CI, 1.50-1.89) and was lower than that for offspring of severely obese mothers with insulin-treated pregestational diabetes (HR, 4.20; 95% CI, 2.67-6.59). However, antidepressants were markedly more common among the offspring of severely obese mothers with type 2 diabetes (HR, 6.85; 95% CI, 2.53-18.54) compared with the offspring of severely obese mothers without diabetes (HR, 0.85; 95% CI, 0.31-2.28) (Figure 2C and Table 3). Of note, we could only exclude marked confounding from maternal inpatient psychiatric disorders and maternal purchase of ATC group N05 or N06 drugs (yes or no) during pregnancy, as seen for 1.8% and 5.8% of the mothers, respectively (eTable 1 in the Supplement).

Fourth, in a second sensitivity analysis, we analyzed all the first 2 siblings born to the mothers in an attempt to adjust for familiar risk of the outcome (247 681 mothers). Most of the sibling pairs were concordant for the maternal diabetes (>97%) and BMI group (>50%) exposure (eTable 2 and eTable 3 in the Supplement), which prohibited an exposure-discordance analysis. We estimated the risk of psychiatric disorder and psychotropic medication for the second offspring given their exposure to diabetes without (model 1) and with (model 2) adjustment for the corresponding psychiatric outcome in the older sibling irrespective of the older sibling’s exposure. The effect sizes for the second sibling were similar to those in the whole cohort (Table 2 and Table 3; eTable 4 in the Supplement), and there was no significant difference in effect sizes between model 1 and model 2 (ie, by adjusting for the older sibling’s psychiatric outcome) (eTable 4 in the Supplement).

## Discussion

In this nationwide cohort study in Finland, we found that maternal moderate and severe obesity combined with type 2 diabetes were associated with mood disorders, intellectual disabilities, specific developmental disorders, and ADHD or conduct disorders among their offspring. The association with mood disorders was unique to the joint associations of high BMI and type 2 diabetes, whereas for the other disorders, an association with high BMI alone was also found, albeit with a smaller effect size than the joint association. For mothers with severe obesity, the effect size of type 2 diabetes on their offspring having any *ICD-10* F code diagnosis was between those of mothers with insulin-treated pregestational diabetes and gestational diabetes. For the first time, to our knowledge, we also report that insulin-treated pregestational diabetes in severely obese mothers was associated with mood disorders and intellectual disability among their offspring, with large effect sizes (pointwise HRs of 8.03 and 3.64, respectively), whereas we reported previously that severely obese mothers who had insulin-treated pregestational diabetes had an estimated pointwise HR of 6 for ASD and ADHD or conduct disorders.^21^ For normal-weight mothers, there were no associations of either type 2 diabetes or insulin-treated pregestational diabetes with offspring having an *ICD-10* F code diagnosis. For mothers with severe obesity, gestational diabetes was associated with disorders in the offspring only when we considered any *ICD-10* F code diagnosis. The fact that the joint association of severe obesity with diabetes was stronger than that of either alone might reflect a stronger neural exposure to inflammation, oxidative stress, lipotoxicity, hyperglycemia, and insulin resistance in the former setting. During pregnancy, insulin resistance is gradually increased to ensure adequate carbohydrate supply for the growing fetus, and along with this, postprandial glucose levels, basal and stimulated insulin secretion, and hepatic glucose production are elevated. An obese and/or diabetic state can aggravate these pregnancy-related metabolic changes. For example, low-grade inflammation, insulin resistance, and hyperinsulinemia are commonly seen in a nonpregnant severely obese setting.^8^ The maternal insulin resistance and hyperglycemia further lead to increased placental cytokine release, glucose transfer, and fetal insulin secretion.^9,25^

Specifically, compared with normal-weight mothers without diabetes, the pointwise effect size for mood disorders was 2 to 3 times higher for obese mothers with type 2 diabetes and was 3 to 8 times higher for obese mothers with insulin-treated pregestational diabetes. We found an effect size greater than 3 for ADHD among offspring of severely obese mothers with type 2 diabetes compared with offspring of normal-weight mothers without diabetes, double that of offspring of moderately obese mothers with type 2 diabetes. However, offspring of normal-weight mothers with type 2 diabetes had no increased risk of ADHD. Accordingly, a Norwegian case-control study^26^ of 2 322 657 mothers, most of whom were of normal weight, showed that maternal type 2 diabetes without considering BMI was not associated with offspring ADHD. Conversely, the offspring of mothers with severe obesity who had insulin-treated pregestational diabetes had an approximately 6-time increased risk of ADHD or conduct disorders.^21^ The effect size for offspring of women with severe obesity who had type 2 diabetes to have ASD was 2 times higher than that for offspring of normal-weight mothers without diabetes, which was less than half of that of severely obese mothers with insulin-treated pregestational diabetes.^21^ Likewise, a retrospective cohort study^17^ including 322 323 children born in the United States from 1995 to 2009 found that type 2 diabetes, without considering BMI, yielded an HR for ASD of 1.33 (95% CI, 1.07-1.66). Similarly, the offspring of US mothers with obesity who had nonspecified pregestational diabetes had an increased risk of ASD (HR, 3.9; 95% CI, 1.8-8.7).^19^ In addition, the offspring of severely obese mothers with type 2 diabetes had a more than 2-fold risk for specific developmental disorders compared with the offspring of normal-weight mothers without diabetes, whereas the corresponding effect size for obese mothers with insulin-treated pregestational diabetes was almost 3 times.

One reason that insulin-treated pregestational diabetes had a higher effect size than type 2 diabetes (not treated with insulin) on the risk that offspring will have an *ICD-10* F code diagnosis might be that mothers with insulin-treated pregestational diabetes have poorer glucose control. Also, insulin plays a role in energy metabolism and other aspects of central nervous system function,^27^ and insulin abnormalities may exacerbate cognitive impairment.^28^ In fact, increased peripheral insulin results in insulin resistance, which itself has substantial outcomes on the brain^29^ and has been associated with depression.^30,31,32^ Furthermore, persons with, for example, ADHD and mood disorders have been reported to have an increased risk of developing obesity. Whether this is genetically or solely environmentally underpinned is not clear.^33,34^ However, common genetic risk factors for psychiatric disorders and obesity are known and can thus provide a possible partial explanation for the association of maternal obesity with the increased risk of psychiatric disorders in the offspring of mothers with diabetes.^35,36^ In children with type 1 diabetes, hypoglycemia events are associated with a higher risk of developing ADHD.^37^

Finally, a sensitivity analysis was performed to estimate the risk for psychotropic medication purchase given maternal prepregnancy obesity and diabetes. Our findings supported the associations observed from the *ICD-10 *F code diagnosis–based analyses.

### Limitations

This study has limitations. The analyses were adjusted for possible confounders with different frequencies in the group with type 2 diabetes compared with the group without diabetes (eTable 1 in the Supplement).^38,39,40^ In an attempt to adjust for socioeconomic position, smoking and marital status were adjusted for because they are associated with more sophisticated measures of socioeconomic position^41^; however, the availability of better indicators of socioeconomic position would have been desirable. Furthermore, reliable information on preeclampsia, blood glucose level, and metabolic control was not available. The purchase of glucose-lowering drugs other than insulin, such as ATC group A10B drugs (mainly metformin), during pregnancy was not adjusted for because few mothers purchased such drugs. Also, maternal BMI was obtained at only a single time point; thus, the association with gestational weight gain could not be examined. As discussed already, there is also potential residual confounding from genetic susceptibility, such as maternal and paternal outpatient psychiatric conditions, that were unavailable for this study. We could only exclude marked confounding from maternal inpatient psychiatric disorders and maternal purchase of ATC group N05 or N06 drugs (yes or no) during pregnancy, as seen for 1.8% and 5.8% of the mothers, respectively (eTable 1 in the Supplement). Analysis of exposure-discordant siblings were not possible because of an exposure concordance rate of more than 97% for diabetes (eTable 2 in the Supplement). Still, adjustment for the corresponding psychiatric diagnosis or psychotropic medication purchase outcome in the older sibling did not reduce the risk estimate of the association between diabetes exposure and offspring psychiatric disorder (eTable 4 in the Supplement).

Additional limitations include that, although the oldest birth year cohort could be followed up for 11 years (until 2014), those born at later birth years were followed up for shorter time, reducing the chance of debut of later-onset disorders (Table 1). Between 27.0% and 39.8% of cases were detected for mood disorders, anxieties, eating disorders, intellectual disabilities, and specific developmental disorders, whereas 55.5% to 76.2% of cases were detected for sleeping disorders, ASD, ADHD and conduct disorders, and other behavioral and emotional disorders (*ICD-10* code F98) (Table 1). The risk for offspring psychiatric disorders or medication did not follow the pattern of proportion picked up. Moreover, there was no clear difference in age at diagnosis between those exposed to maternal obesity or diabetes and those not exposed (eTable 5 in the Supplement). Furthermore, because we prioritized sensitivity over specificity in the detection of type 2 diabetes among the mothers, a diagnosis with *ICD-10* code E14, or a purchase of an ATC group A10B drug, before pregnancy was enough to be classified as having type 2 diabetes. Thus, some mothers with true unspecified diabetes or prediabetes were probably included in the type 2 diabetes group. However, 90% had a type 2 diabetes diagnosis according to *ICD-10* codes O24.1 and E11. In addition, we did not adjust for multiple testing.

## Conclusions

Maternal diabetes, combined with severe obesity, was associated with overall offspring neuropsychiatric disorder risk in a hierarchical order, with insulin-treated pregestational diabetes associated with the greatest risk, followed by type 2 diabetes and then gestational diabetes, where insulin treatment was present only in the group with insulin-treated pregestational diabetes. These risk estimates were larger than those for either maternal severe obesity or diabetes alone. The largest risk estimates were found for offspring mood disorders, ASD, and ADHD or conduct disorders. Further studies with longer offspring follow-up time and exploration of biological mechanisms are required.

## Notes

Supplement. **eAppendix.** Supplemental Methods. **eTable 1. **Demographic Characteristics of Offspring and Their Mothers (N = 647 099 Studied Births out of the Total 649 043 Births) **eTable 2. **All Singleton Sibling Pairs (n=247 681) With the Same Mother in the Birth Cohorts 2004-2014: Exposures During Pregnancy to Maternal Diabetes Mellitus Diagnoses **eTable 3. **All Singleton Sibling Pairs (n=247 681) With the Same Mother in the Birth Cohorts 2004-2014: Exposures During Pregnancy to Maternal Underweight, Overweight, and Obesity **eTable 4. **Sibling Pair Analysis in All Mothers With a Singleton Sibling Pair (n=247 681) 2004-2014: Adjusted Cox Hazard Ratios (HR) and 95% Confidence Intervals (CI) for the Outcomes in the Second Child Being (i) Any Psychiatric Disorder and ADHD/Conduct Disorders (F90-91), and (ii) Any Psychotropic Medication and Stimulants (N06B) From Birth Until 2014 **eTable 5. **Age at Onset [Years] for Births 2004-2009 Considering F Diagnoses After Birth Until 2014 in Different Maternal Exposure Groups (Median, 25th and 75th Percentiles)
