# Supplementary material for: Associations of Different Types of Maternal Diabetes and Body Mass Index With Offspring Psychiatric Disorders
Source: JAMA Netw Open. 2020 Feb 7;3(2):e1920787. doi: 10.1001/jamanetworkopen.2019.20787 (PMC12578498; doi:10.1001/jamanetworkopen.2019.20787)
Supplement: Supplement. — eAppendix. Supplemental Methods. eTable 1. Demographic Characteristics of Offspring and Their Mothers (N = 647 099 Studied Births out of the Total 649 043 Births) eTable 2. All Singleton Sibling Pairs (n=247 681) With the Same Mother in the Birth Cohorts 2004-2014: Exposures During Pregnancy to Maternal Diabetes Mellitus Diagnoses eTable 3. All Singleton Sibling Pairs (n=247 681) With the Same Mother in the Birth Cohorts 2004-2014: Exposures During Pregnancy to Maternal Underweight, Overweight, and Obesity eTable 4. Sibling Pair Analysis in All Mothers With a Singleton Sibling Pair (n=247 681) 2004-2014: Adjusted Cox Hazard Ratios (HR) and 95% Confidence Intervals (CI) for the Outcomes in the Second Child Being (i) Any Psychiatric Disorder and ADHD/Conduct Disorders (F90-91), and (ii) Any Psychotropic Medication and Stimulants (N06B) From Birth Until 2014 eTable 5. Age at Onset [Years] for Births 2004-2009 Considering F Diagnoses After Birth Until 2014 in Different Maternal Exposure Groups (Median, 25th and 75th Percentiles) [file jamanetwopen-e1920787-s001.pdf]

Supplementary Online Content

Kong L, Nilsson IAK, Brismar K, Gissler M, Lavebratt C. Associations of different types of maternal diabetes and body mass index with offspring psychiatric disorders. *JAMA Netw Open*. 2020;3(2):e1920787. doi:10.110/jamanetworkopen.2019.20787

eAppendix. Supplemental Methods.

eTable 1. Demographic Characteristics of Offspring and Their Mothers (N = 647 099 Studied Births Out of the Total 649 043 Births)

eTable 2. All Singleton Sibling Pairs (n=247 681) With the Same Mother in the Birth Cohorts 2004-2014: Exposures During Pregnancy to Maternal Diabetes Mellitus Diagnoses

eTable 3. All Singleton Sibling Pairs (n=247 681) With the Same Mother in the Birth Cohorts 2004-2014: Exposures During Pregnancy to Maternal Underweight, Overweight, and Obesity

eTable 4. Sibling Pair Analysis in All Mothers With a Singleton Sibling Pair (n=247 681) 2004-2014: Adjusted Cox Hazard Ratios (HR) and 95% Confidence Intervals (CI) for the Outcomes in the Second Child Being (i) Any Psychiatric Disorder and ADHD/Conduct Disorders (F90-91), and (ii) Any Psychotropic Medication and Stimulants (N06B) From Birth Until 2014

eTable 5. Age at Onset [Years] for Births 2004-2009 Considering F Diagnoses After Birth Until 2014 in Different Maternal Exposure Groups (Median, 25th and 75th Percentiles)

This supplementary material has been provided by the authors to give readers additional information about their work.

eAppendix. Supplemental Methods

Data sources

The Medical Birth Register (MBR), the Register of Congenital Malformations and the Register on Induced Abortions are currently stored at the Finnish National Institute for Health and Welfare (THL). MBR contains information since 1987 on all live births and stillbirths in Finland with a gestational age of ≥ 22 weeks or a birth weight ≥ 500 g. The Finnish Register on Reimbursement Drugs (RRD, KELA) is kept by the National Social Insurance Institution (SII). Since 1994, RRD has registered all reimbursed drug prescriptions (ATC-code) that were dispensed at pharmacies. The Finnish Care Registers for Health Care (HILMO) contains data on all hospital in-patient treatments (since 1969) as well as out-patient treatments by physicians in specialized care (since 1998), and covers adult psychiatric diagnoses well, according to validation studies[1], while there is only one validation study on pediatric psychiatric diagnoses in HILMO, and that one reported good validity for pediatric Autism spectrum disorder[2].

Reference

1. Sund R. Quality of the Finnish Hospital Discharge Register: a systematic review. Scand J Public Health 2012;40(6):505-515.
2. Lampi K, Sourander A, Gissler M, et al. Brief report: validity of Finnish registry-based diagnoses of autism with the ADI-R. Acta Paediatr 2010;99(9):1425-1428.

© 2020 Kong L et al. *JAMA Network Open.*

eTable 1. Demographic Characteristics of Offspring and Their Mothers (N = 647 099 Studied Births Out of the Total 649 043 Births)

Variables	No diabetes N=541 133	PGDM-insulin[a] N=4000	T2DM[b] N=3724	GDM N=98 242
	No. (%)	No. (%)	No. (%)	No. (%)
**Offspring birth year**				
2004	47,295 (8.7)	293 (7.3)	440 (11.8)	9,310 (9.5)
2005	47,811 (8.8)	307 (7.7)	384 (10.3)	8,496 (8.6)
2006	49,483 (9.1)	349 (8.7)	416 (11.2)	8,125 (8.3)
2007	49,241 (9.1)	349 (8.7)	408 (11.0)	8,631 (8.8)
2008	49,749 (9.2)	325 (8.1)	365 (9.8)	8,292 (8.4)
2009	50,961 (9.4)	369 (9.2)	353 (9.5)	8,686 (8.8)
2010	51,506 (9.5)	382 (9.6)	323 (8.7)	8,913 (9.1)
2011	49,905 (9.2)	371 (9.3)	322 (8.6)	9,418 (9.6)
2012	49,529 (9.2)	400 (10.0)	258 (6.9)	9,435 (9.6)
2013	48,063 (8.9)	429 (10.7)	247 (6.6)	9,583 (9.8)
2014	47,590 (8.8)	426 (10.7)	208 (5.6)	9,353 (9.5)
**Offspring sex**				
Boy	276,134 (51.0)	2,025 (50.6)	1,929 (51.8)	50,781 (51.7)
Girl	264,999 (49.0)	1,975 (49.4)	1,795 (48.2)	47,461 (48.3)
**Number of fetuses**				
1	524,319 (96.9)	3,892 (97.3)	3,668 (98.5)	96,725 (98.5)
2	16,490 (3.0)	105 (2.6)	55 (1.5)	1,506 (1.5)
3	320 (0.1)	3 (0.1)	1 (0.0)	11 (0.0)
4	4 (0.0)	0 (0.0)	0 (0.0)	0 (0.0)
**Mode of delivery**				
Vaginal	409,382 (75.7)	1,232 (30.8)	2,418 (64.9)	70,633 (71.9)
Instrumental	45,932 (8.5)	265 (6.6)	255 (6.8)	7,609 (7.7)
Planned CS	34,235 (6.3)	1,239 (31.0)	464 (12.5)	8,013 (8.2)
Other CS	51,199 (9.5)	1,261 (31.5)	586 (15.7)	11,954 (12.2)
Missing	385 (0.0)	3 (0.1)	1 (0.0)	33 (0.0)
**Maternal age (years)**				
13-19	13,579 (2.5)	89 (2.2)	55 (1.5)	1,409 (1.4)
20–24	87,164 (16.1)	627 (15.7)	407 (10.9)	11,953 (12.2)
25–29	173,816 (32.1)	1,271 (31.8)	944 (25.3)	28,594 (29.1)
30–34	170,246 (31.5)	1,220 (30.5)	1,175 (31.6)	31,923 (32.5)
35–55	96,328 (17.8)	793 (19.8)	1,143 (30.7)	24,363 (24.8)
**Parity**				
0	231,845 (42.8)	1,778 (44.5)	1,219 (32.7)	35,682 (36.3)
1	182,974 (33.8)	1,370 (34.3)	1,091 (29.3)	31,982 (32.6)
2	77,040 (14.2)	523 (13.1)	683 (18.3)	16,881 (17.2)
3	25,539 (4.7)	179 (4.5)	307 (8.2)	6,993 (7.1)
4 or more	23,307 (4.3)	148 (3.7)	421 (11.3)	6,658 (6.8)
Missing	428 (0.1)	2 (0.1)	3 (0.1)	46 (0.1)
**Mother’s marital status**				
Married	491,878 (90.9)	3,592 (89.8)	3,356 (90.1)	90,480 (92.1)
Unmarried	49,255 (9.1)	408 (10.2)	368 (9.9)	7,762 (7.9)

© 2020 Kong L et al. *JAMA Network Open.*

eTable 1. (Continued)

Variables	No diabetes N=541 133	PGDM-insulin[a] N=4000	T2DM[b] N=3724	GDM N=98 242
	No. (%)	No. (%)	No. (%)	No. (%)
**Mother's country of birth**				
Finland	490,851 (90.7)	3,818 (95.5)	3,271 (87.8)	88,372 (90.0)
Other	48,711 (9.0)	182 (4.6)	443 (11.9)	9,765 (9.9)
Missing	1,571 (0.3)	0 (0.0)	10 (0.2)	105 (0.1)
**Maternal smoking**				
No	447,971 (82.8)	3,204 (80.1)	2,880 (77.3)	79,727 (81.2)
Stopped in T1 trimester	26,816 (5.0)	236 (5.9)	178 (4.8)	5,605 (5.7)
Continued	53,505 (9.9)	460 (11.5)	540 (14.5)	10,423 (10.6)
Missing	12,841 (2.4)	100 (2.5)	126 (3.4)	2,487 (2.5)
**Maternal BMI [kg/m^2]**				
<18.5	21,771 (4.0)	65 (1.6)	29 (0.8)	1,144 (1.2)
18.5–24	347,888 (64.3)	1,840 (46.0)	895 (24.0)	32,423 (33.0)
25–29	101,288 (18.7)	1,068 (26.7)	853 (22.9)	30,724 (31.3)
30–34	30,591 (5.7)	475 (11.9)	803 (21.6)	17,790 (18.1)
35–	10,911 (2.0)	349 (8.7)	937 (25.2)	11,478 (11.7)
Missing	28,684 (5.3)	203 (5.1)	207 (5.6)	4,683 (4.8)
**Maternal psychiatric diagnosis**				
No	531,930 (98.3)	3,904 (97.6)	3,568 (95.8)	96,016 (97.7)
Yes	9,203 (1.7)	96 (2.4)	156 (4.2)	2,226 (2.3)
**Maternal psychotropic medication use (N05/N06)**				
No	511,342 (94.5)	3,633 (90.8)	3,335 (89.6)	91,265 (92.9)
Yes	29,791 (5.5)	367 (9.2)	389 (10.4)	6,977 (7.1)
**Birth weight (mean, SD)**	3,485 ± 586	3,645 ± 774	3,619 ± 683	3,625 ± 584
**Gestational age (mean,SD)**	279.4 ± 24.6	261.0 ± 29.0	273.5 ± 23.2	278.4 ± 21.9

Abbreviations: CS, caesarean section. BMI, Body mass index [kg/m^2]. PGDM-insulin, pre-gestational diabetes mellitus with insulin treatment according to purchase of insulin prior to pregnancy. T2DM, pre-gestational type 2 diabetes mellitus (ICD-10 E11, E14, O24.1 and/or ATC A10B) without purchase of insulin before or during pregnancy. GDM, gestational diabetes mellitus according to an ICD–10 O24.4 diagnosis without purchase of insulin before or during pregnancy. No DM, none of PGDM-insulin, T2DM or GDM. T1, trimester 1. N=only 647 099 out of the 649 043 births were studied because births with insulin treatment during pregnancy in the categories No DM, T2DM and GDM were excluded.

[a] Mothers with PGDM-insulin were excluded from the T2DM and GDM groups.

[b] Mothers with T2DM were excluded from the GDM group.

© 2020 Kong L et al. *JAMA Network Open.*

eTable 2. All Singleton Sibling Pairs (n=247 681) With the Same Mother in the Birth Cohorts 2004-2014: Exposures During Pregnancy to Maternal Diabetes Mellitus Diagnoses

Diabetes exposure	No DM[b]	PGDM-insulin[b]	T2DM[b]	GDM[b]
**No DM[a]**	202,973 (99.4%)	6 (0%)	14 (0%)	1,134 (0.6%)
**PGDM-insulin[a]**	0 (0%)	722 (99.4%)	4 (0.6%)	0 (0%)
**T2DM[a]**	24 (1.1%)	11 (0.5%)	2,085 (97.6%)	16 (0.7%)
**GDM[a]**	1,126 (2.8%)	38 (0.1%)	19 (0%)	39,509 (97.1%)

[a] Maternal diabetes exposure in first pregnancy, [b] Maternal diabetes exposure in second pregnancy

Abbreviation: PGDM-insulin, pre-gestational diabetes with purchase of insulin prior to pregnancy. T2DM, pre-gestational type 2 diabetes mellitus (ICD-10 E11, E14, O24.1 and/or ATC A10B) without insulin purchase before or during pregnancy. GDM, gestational diabetes mellitus according to an ICD–10 O24.4 diagnoses without purchase of insulin before or during pregnancy. No DM, none of PGDM-insulin, T2DM or GDM.

© 2020 Kong L et al. *JAMA Network Open.*

eTable 3. All Singleton Sibling Pairs (n=247 681) With the Same Mother in the Birth Cohorts 2004-2014: Exposures During Pregnancy to Maternal Underweight, Overweight, and Obesity

BMI exposure	Underweight[b]	Normal weight[b]	Overweight[b]	Obesity[b]	Severe obesity[b]	Missing[b]
**Underweight[a]**	5,106 (51.5%)	4,549 (45.9%)	53 (0.5%)	11 (0.1%)	8 (0.1%)	181 (1.8%)
**Normal weight[a]**	2,471 (1.6%)	124,889 (82.6%)	20,184 (13.4%)	976 (0.6%)	78 (0.1%)	2,538 (1.7%)
**Overweight[a]**	16 (0%)	7,118 (15.1%)	29,873 (63.2%)	8,708 (18.4%)	682 (1.4%)	863 (1.8%)
**Obesity[a]**	7 (0%)	269 (1.6%)	2,812 (17%)	9,635 (58.3%)	3,481 (21.1%)	325 (2.0%)
**Severe obesity[a]**	3 (0%)	32 (0.4%)	155 (2.1%)	1,114 (15.3%)	5,831 (80.0%)	151 (2.1%)
**Missing[a]**	512 (3.3%)	8,760 (56.3%)	2,930 (18.8%)	1,129 (7.3%)	487 (3.1%)	1,744 (11.2%)

[a] Maternal pre-pregnancy body mass index (BMI) exposure in first pregnancy, [b] Maternal BMI exposure in second pregnancy.

BMI was categorized as: underweight (BMI<18.5 kg/m^2), normal weight (18.5≤BMI<25 kg/m^2), overweight (25≤BMI<30 kg/m^2), moderately obese (30≤BMI<35 kg/m^2), severely obese (BMI≥35 kg/m^2).

© 2020 Kong L et al. *JAMA Network Open.*

eTable 4. Sibling Pair Analysis in All Mothers With a Singleton Sibling Pair (n=247 681) 2004-2014: Adjusted Cox Hazard Ratios (HR) and 95% Confidence Intervals (CI) for the Outcomes in the Second Child Being (i) Any Psychiatric Disorder and ADHD/Conduct Disorders (F90-91), and (ii) Any Psychotropic Medication and Stimulants (N06B) From Birth Until 2014

Exposure for second child irrespective of exposure for first child	Any F diagnosis		ADHD/ conduct disorders		Any medication (N05 or N06)		Stimulants (N06B)	
	HR	95% CI	HR	95% CI	HR	95% CI	HR	95% CI
**Model 1**								
No DM								
BMI < 25	1.00	NA	1.00	NA	1.00	NA	1.00	NA
BMI ≥ 25	1.18	1.12-1.24	1.18	0.99-1.41	1.20	1.10-1.31	1.32	1.07-1.62
PGDM-insulin								
All BMI	0.97	0.69-1.38	1.51	0.56-4.12	1.35	0.82-2.23	2.68	1.08-6.66
T2DM								
BMI < 30	1.40	1.09-1.80	1.84	0.87-3.90	1.09	0.69-1.74	1.89	0.78-4.61
BMI 30-34	2.01	1.43-2.83	2.19	0.70-6.85	0.85	0.35-2.04	1.04	0.15-7.46
BMI ≥ 35	1.94	1.38-2.74	3.91	1.61-9.47	3.32	2.11-5.23	4.55	1.69-12.27
GDM								
BMI < 30	1.19	1.11-1.28	1.18	0.92-1.50	1.11	0.99-1.25	1.04	0.76-1.41
BMI 30-34	1.43	1.28-1.60	1.66	1.18-2.35	1.35	1.12-1.62	1.96	1.34-2.88
BMI ≥ 35	1.79	1.57-2.03	2.72	1.91-3.89	1.58	1.26-1.97	3.11	2.05-4.73
**Model 2**								
No DM								
BMI < 25	1.00	NA	1.00	NA	1.00	NA	1.00	NA
BMI ≥ 25	1.15	1.09-1.21	1.13	0.95-1.35	1.19	1.09-1.29	1.27	1.03-1.56
PGDM-insulin								
All BMI	0.92	0.65-1.31	1.60	0.59-4.34	1.36	0.83-2.24	2.76	1.11-6.86
T2DM								
BMI < 30	1.31	1.02-1.69	1.76	0.83-3.74	1.05	0.66-1.68	1.69	0.69-4.12
BMI 30-34	1.79	1.27-2.52	2.17	0.70-6.78	0.82	0.34-1.99	1.02	0.14-7.26
BMI ≥ 35	1.77	1.26-2.50	3.60	1.48-8.76	3.21	2.04-5.06	4.46	1.65-12.07
GDM								
BMI < 30	1.17	1.09-1.25	1.13	0.89-1.44	1.11	0.98-1.25	1.00	0.73-1.37
BMI 30-34	1.36	1.22-1.52	1.54	1.09-2.18	1.32	1.10-1.58	1.78	1.21-2.61
BMI ≥ 35	1.68	1.48-1-90	2.49	1.75-3.57	1.53	1.22-1.91	2.57	1.69-3.92

Abbreviations: HRs, hazard ratios. ADHD, attention deficit hyperactivity disorder. N06B, stimulants. BMI, body mass index [kg/m^2]. PGDM-insulin, pre-gestational diabetes with purchase of insulin prior to pregnancy. T2DM, pre-gestational type 2 diabetes mellitus (ICD-10 E11, E14, O24.1 and/or ATC A10B) without insulin purchase before or during pregnancy. GDM, gestational diabetes mellitus according to an ICD–10 O24.4 diagnoses without purchase of insulin before or during pregnancy. No DM, none of PGDM-

© 2020 Kong L et al. *JAMA Network Open.*

insulin, T2DM or GDM. NA, not available. For Any F-diagnosis the birth cohorts 2004-2014 were used. For ADHD the birth cohorts 2004-2012 were used. All were followed up until 2014.

Model 1: Adjusted for the following variables at the second pregnancy: maternal age, parity, maternal smoking during pregnancy [yes/no], mother unmarried [yes/no], mother born elsewhere than Finland [yes/no], caesarean section [yes/no], mother's inpatient care due to mental health disorders [yes/no], mother's purchase of psychotropic drugs (N05 or N06) during pregnancy [yes/no], mother's diagnoses related to systemic inflammatory disorders [yes/no], multiple birth [yes/no], offspring sex, perinatal health problems (birth weight < 2500 grams, gestational age < 37 weeks or small for gestational age according to Finnish sex-specific standards) [yes/no].

Model 2: Adjusted for the variables in Model 1 as well as the second (youngest) child's outcome in the first (oldest) child, i.e. any F diagnosis, any psychotropic medication (N05 or N06), ADHD/conduct disorders (F90-91) or stimulants (N06B), respectively, in the first child.

© 2020 Kong L et al. *JAMA Network Open.*

eTable 5. Age at Onset [Years] for Births 2004-2009 Considering F Diagnoses After Birth Until 2014 in Different Maternal Exposure Groups (Median, 25th and 75th Percentiles)

ICD-10 codes	No DM, BMI<25 kg/m^2	No DM, BMI>29 kg/m^2	PGDM-insulin/ T2DM, BMI>29 kg/m^2	GDM, BMI>29 kg/m^2
	Median (25th, 75th)	Median (25th, 75th)	Median (25th, 75th)	Median (25th, 75th)
F30-39, F92	5.1 (3.5, 6.5)	5.0 (3.6, 6.4)	4.7 (3.3, 6.5)	5.2 (3.7, 6.7)
F40-43, F93	6.7 (5.3, 8.0)	6.4 (4.8, 7.7)	6.2 (4.6, 8.0)	6.7 (5.4, 8.0)
F50	5.9 (4.5, 7.5)	5.8 (4.5, 7.3)	6.0 (4.1, 8.3)	6.1 (4.6, 7.9)
F51	4.2 (1.3, 6.8)	2.2 (1.2, 4.3)	NA	2.3 (1.1, 4.1)
F60-69	1.0 (0.7, 2.6)	1.2 (0.6, 2.8)	1.6 (0.8, 2.7)	1.3 (0.8, 3.1)
F70-79	6.3 (4.6, 8.7)	6.2 (2.1, 7.9)	NA	5.4 (3.6, 6.7)
F80–83	3.8 (2.6, 5.9)	3.8 (2.9, 5.6)	3.9 (2.8, 4.9)	3.9 (2.8, 5.1)
F84	4.7 (3.5, 5.8)	4.5 (3.5, 5.7)	4.5 (3.2, 5.8)	4.7 (3.6, 6.0)
F90–91	4.3 (3.1, 6.0)	3.8 (3.1, 5.4)	4.3 (3.7, 6.1)	4.3 (3.1, 5.8)
F98	6.2 (4.8, 7.6)	5.9 (4.5, 7.3)	6.0 (4.5, 7.3)	6.4 (5.0, 7.6)

Abbreviations: ICD-10. International Statistical Classification of Diseases and Related Health Problems 10th Revision. BMI, body mass index [kg/m^2]. PGDM-insulin, pre-gestational diabetes with purchase of insulin prior to pregnancy. T2DM, pre-gestational type 2 diabetes mellitus (ICD-10 E11, E14, O24.1 and/or ATC A10B) without insulin purchase before or during pregnancy. GDM, gestational diabetes mellitus according to an ICD–10 O24.4 diagnoses without purchase of insulin before or during pregnancy. No DM, none of PGDM-insulin, T2DM or GDM. NA, not available.

© 2020 Kong L et al. *JAMA Network Open.*
